# The National Health Week

**Published:** 1912-04-27

**Authors:** 


### The Hospital

The Workers' Newspaper of Administrative Medicine and Institutional Life, Administration, National Insurance and Health.

No. 1346, Vol. LII. SATURDAY, APRIL 27, 1912. PRICE ONE PENNY.

### THE NATIONAL HEALTH WEEK.

The National Health Week, which is to be celebrated this ensuing week, is a hopeful sign of the times in which we live, as well as an indication of the way in which the Agenda Club is living up to its name. The members of this organisation are drawn, happily, from all parties and all creeds; and the inspiration which prompted the club to concern itself with national health is one which can elicit nothing but praise from all quarters. At the same time it may be doubted whether the reception by the community of the scheme which the enthusiasm and energy of the Agenda Club's members is recommending to public notice would have been anything like so interested and so cordial five or ten years ago as it is to-day. The nation has awakened to the fact that large sections of it are born and reared in conditions which militate so seriously against health of mind and body that the safety of the State is thereby seriously endangered. *Salus populi suprema lex* is a maxim whose truth and tremendous import are gradually receiving the recognition which has long been denied to them. Politicians may differ over the exact steps which may best be taken to minimise and eventually to avert the evils of overcrowding, of insanitation, of preventable disease, and the other offences against a hygienic mode of life which are rampant in our midst. But all thinking men are beginning to realise the pressing nature of the problems of health and life; and with time and patience the unthinking and careless majority will be influenced, perhaps unconsciously, by the more intelligent.

For bringing about the change in public opinion about matters of public health from apathy to interest, no single individual claims the credit. A large share of it, indubitably, must be allotted to the medical profession as a whole, whose preaching and practice for the last generation have steadily improved and have been leavening the lump of general indifference. The institutional world has also, without question, been instrumental in diffusing light into the darkness of general ignorance among the working-classes. The educational influence of the voluntary hospital, the consumption sanatoria, and the Poor-law infirmary upon those whom accident or illness has brought within their walls, has often received acknowledgment in the columns of The Hospital; and indeed it would be difficult to overestimate the value of this influence. The policy of the educational authorities up and down the kingdom is another factor which contributes a by no means negligible quota to the sum of general enlightenment; and it must be confessed that in this respect the working-classes have had more advantages than many of those whose parents pay for their education. Still, there yet remains much to be done before the instruction of the young in the laws of health can be considered to be really satisfactory or commensurate to the vital needs of the nation at large.

The programme of the Agenda Club is, therefore, one which it is incumbent on all to further and assist in every possible way. Its details are too numerous to be set forth here, but can be obtained by those who are interested from the National Health Week Committee, Agenda Club, 28 Fleet Street, E.C.

As an instance of what it may be possible to do, an excellent letter from the Headmaster of Rugby to the *Times* may be read with profit. In it he announces his intention to preach to his boys on Sunday, May 5, on the ethical and religious aspects of health and hygiene; and he invites others similarly situated to do the same. He proceeds to suggest that in the evening it may be possible for the school medical officer to lecture on the national and social aspect of the fight against disease and its allies. The medical officer at Rugby is thus offered an opportunity of which he will, we feel certain, take full advantage; and the suggestion is so excellent that the publicity which it has received will surely lead to its widespread adoption. If to offer an amendment to the scheme would not seem ungracious, it might be suggested that an even better plan for securing the ear of a compulsory congregation would be for the headmaster to vacate his morning pulpit in favour of his medical officer, who would thus be far more certain of an attentive audience. This is not to decry the "ethical and religious aspects" of health and hygiene, to the value of which we are as fully alive as the Headmaster of Rugby. Nor is it only at the big public schools that we would see the suggestion carried out. Private schools and elementary schools have, it is true, no medical officer attached to them quite like (most of) the public schools have; but it is quite possible for those in charge of them to seek the counsel of some neighbouring practitioner of repute. Aid will not be refused them, unless our knowledge of the medical profession is greatly at fault. It is by catching Britain's future citizens young and impressionable that the next generation may be brought up more enlightened in matters of hygiene than the present or the last one; and it is on such enlightenment that the future prosperity of the kingdom depends. Of that next generation the Agenda Club deserves well, and we trust it will not relax its plan of campaign after the end of its National Health Week.

